# Cardiomyocyte substructure reverts to an immature phenotype during heart failure

**DOI:** 10.1113/JP277273

**Published:** 2019-02-27

**Authors:** D. B. Lipsett, M. Frisk, J. M. Aronsen, E. S. Nordén, O. R. Buonarati, A. Cataliotti, J. W. Hell, I. Sjaastad, G. Christensen, W. E. Louch

**Affiliations:** ^1^ Institute for Experimental Medical Research Oslo University Hospital and University of Oslo Oslo Norway; ^2^ KG Jebsen Center for Cardiac Research University of Oslo Oslo Norway; ^3^ Bjørknes College Oslo Norway; ^4^ Department of Pharmacology University of California Davis Davis CA USA

**Keywords:** development, heart failure, t‐tubule, dyad, calcium homeostasis

## Abstract

**Key points:**

- As reactivation of the fetal gene program has been implicated in pathological remodelling during heart failure (HF), we examined whether cardiomyocyte subcellular structure and function revert to an immature phenotype during this disease.
- Surface and internal membrane structures appeared gradually during development, and returned to a juvenile state during HF. Similarly, dyadic junctions between the cell membrane and sarcoplasmic reticulum were progressively ‘packed’ with L‐type Ca^2+^ channels and ryanodine receptors during development, and ‘unpacked’ during HF.
- Despite similarities in subcellular structure, dyads were observed to be functional from early developmental stages, but exhibited an impaired ability to release Ca^2+^ in failing cardiomyocytes.
- Thus, while immature and failing cardiomyocytes share similarities in subcellular structure, these do not fully account for the marked impairment of Ca^2+^ homeostasis observed in HF.

**Abstract:**

Reactivation of the fetal gene programme has been implicated as a driver of pathological cardiac remodelling. Here we examined whether pathological remodelling of cardiomyocyte substructure and function during heart failure (HF) reflects a reversion to an immature phenotype. Using scanning electron microscopy, we observed that Z‐grooves and t‐tubule openings at the cell surface appeared gradually during cardiac development, and disappeared during HF. Confocal and super‐resolution imaging within the cell interior revealed similar structural parallels; disorganization of t‐tubules in failing cells was strikingly reminiscent of the late stages of postnatal development, with fewer transverse elements and a high proportion of longitudinal tubules. Ryanodine receptors (RyRs) were observed to be laid down in advance of developing t‐tubules and similarly ‘orphaned’ in HF, although RyR distribution along Z‐lines was relatively sparse. Indeed, nanoscale imaging revealed coordinated packing of L‐type Ca^2+^ channels and RyRs into dyadic junctions during development, and orderly unpacking during HF. These findings support a ‘last in, first out’ paradigm, as the latest stages of dyadic structural development are reversed during disease. Paired imaging of t‐tubules and Ca^2+^ showed that the disorganized arrangement of dyads in immature and failing cells promoted desynchronized and slowed Ca^2+^ release in these two states. However, while developing cells exhibited efficient triggering of Ca^2+^ release at newly formed dyads, dyadic function was impaired in failing cells despite similar organization of Ca^2+^ handling proteins. Thus, pathologically deficient Ca^2+^ homeostasis during HF is only partly linked to the re‐emergence of immature subcellular structure, and additionally reflects lost dyadic functionality.

## Key points

- As reactivation of the fetal gene program has been implicated in pathological remodelling during heart failure (HF), we examined whether cardiomyocyte subcellular structure and function revert to an immature phenotype during this disease.
- Surface and internal membrane structures appeared gradually during development, and returned to a juvenile state during HF. Similarly, dyadic junctions between the cell membrane and sarcoplasmic reticulum were progressively ‘packed’ with L‐type Ca^2+^ channels and ryanodine receptors during development, and ‘unpacked’ during HF.
- Despite similarities in subcellular structure, dyads were observed to be functional from early developmental stages, but exhibited an impaired ability to release Ca^2+^ in failing cardiomyocytes.
- Thus, while immature and failing cardiomyocytes share similarities in subcellular structure, these do not fully account for the marked impairment of Ca^2+^ homeostasis observed in HF.

## Introduction

Contractility of cardiac muscle cells, or cardiomyocytes, is highly dependent on efficient cycling of Ca^2+^ across cellular membranes. During the action potential, opening of L‐type Ca^2+^ channels (LTCCs) in the surface sarcolemma triggers release of Ca^2+^ from ryanodine receptors (RyRs) in the sarcoplasmic reticulum (for review, see Bers, 2002). Efficient, coordinated triggering of Ca^2+^ release is highly dependent on close localization of these proteins at dyadic junctions. In healthy adult cardiomyocytes, a network of membrane invaginations called t‐tubules enables the formation of dyads deep within the cell interior. These structures appear to play a crucial role in pathology, as remodelling of the t‐tubule network has been observed in various types of cardiac disease (for review see Louch *et al*. 2010; Guo *et al*. 2013; Manfra *et al*. 2017). We and others have shown that considerable reorganization of the t‐tubule network during heart failure (HF) is linked to slower, more dyssynchronous Ca^2+^ release and weaker contraction (Louch *et al*. 2006; Heinzel *et al*. 2008). Initial studies suggested that delayed Ca^2+^ release in failing cells was the result of RyRs that had become orphaned due to disruption to t‐tubules (Song *et al*. 2006). More recent studies, however, have suggested that the function of individual t‐tubules is also affected in failing cells. Indeed, simultaneous multisite voltage and Ca^2+^ recordings found that individual t‐tubules in failing rat cardiomyocytes were not depolarized and that Ca^2+^ release was delayed at these sites (Crocini *et al*. 2014). Additionally, several studies have reported decreased Ca^2+^ current density within the t‐tubules of failing cardiomyocytes (Bryant *et al*. 2015, 2018). These studies suggest that pathological alterations to t‐tubules during HF may not be limited to their structure.

The precise mechanisms underlying pathological remodelling of t‐tubules remain obscure; however, reactivation of gene programmes involved in early cardiac development has been implicated in several other aspects of pathological remodelling during HF (for review see Rajabi *et al*. 2007). We hypothesized here that remodelling of cardiomyocyte substructure during HF exhibits a structural and functional reversion to an immature phenotype. Building on previous work showing that t‐tubules form gradually after birth in rodents (Ziman *et al*. 2010; Hamaguchi *et al*. 2013), we employed advanced imaging studies to show that progressive organization of both the surface membrane and t‐tubule network during this period includes coordinated packing of LTCCs and RyRs into dyads. We observed that HF development reflects a reversal of this process as dyads unpack, and the well‐organized structure of the cell surface and interior is lost. Despite structural resemblance between these two states, stark functional differences were observed; t‐tubules were found to be functional shortly after their growth in the developing heart, but functionally deficient in HF.

## Methods

### Ethical approval

All experiments were approved by the Norwegian National Animal Research Authority (project license no. FOTS 5982, 7786) and performed in accordance with the National Institute of Health guidelines (NIH publication No. 85‐23, revised 2011) and European Directive 2010/63/EU.

Animals were purchased from Janvier Labs (Le Genest‐Saint‐Isle, France) and housed in temperature‐regulated rooms with a 12 h light–dark cycle. Pregnant female Wistar rats were housed (one per cage) with access to food and water *ad libitum*. Following birth, pups were collected at 15, 17.5, 20 and 30 days after birth and heart tissue was extracted while under anaesthesia (5% isofluorane, 95% O_2_) for various experimental purposes. Eight‐ to ten‐week‐old rats were considered adults.

Myocardial infarction was induced in male Wistar rats (∼300 g, 10 weeks old) as described previously (Aronsen *et al*. 2017). In short, rats were intubated and kept under anaesthesia with a mixture of 65% N_2_O, 32% O_2_, and 2–3% isoflurane. Myocardial infarction was induced via ligation of the left anterior descending coronary artery and HF progression was assessed via echocardiography and magnetic resonance imaging after 6 weeks. SHAM‐operated controls underwent the same procedure minus coronary ligation. Buprenorphine was used for analgesia during postoperative care.

The authors understand the ethical principles maintained by *The Journal of Physiology*, and the work complies with *The Journal*’s animal ethics checklist.

### Cell isolation

Individual cardiomyocytes were isolated according to a previously described protocol (Hodne *et al*. 2017). Briefly, animals were injected with 1000 IU/kg heparin (LEO Pharma A/S, Ballerup, Denmark) then anaesthetized by inhalation of a mixture of 5% isoflurane and 95% O_2_. Hearts were extracted and immediately placed in ice‐cold, oxygenated isolation buffer containing (in mmol/l): 130 NaCl, 25 Hepes, 0.5 MgCl_2_, 5.4 KCl, 0.4 NaH_2_PO_4_, 22 glucose. Hearts were quickly cannulated via the aorta and retrogradely perfused with 37°C isolation buffer at a rate of 3 ml/min. Once clear of blood, the myocardium was digested for a period of 5–12 min (depending on age and size of heart) by perfusion of 0.3–1.8 mg/ml of collagenase, type 2 (Worthington Biomedical Corp., Lakewood, NJ, USA) in oxygenated isolation buffer supplemented with 10 μmol/l Ca^2+^. Following digestion, the ventricles were trimmed away from the remaining tissue and submerged in collagenase buffer. Right ventricular tissue was removed and the remaining left ventricle was dissected into approximately 3 mm^3^ chunks. Tissue was gently teased apart with forceps then transferred to a conical tube containing 0.2 mg DNase (Worthington) and 20 mg/ml BSA (Sigma‐Aldrich, St Louis, MO, USA) in dH_2_O. A plastic Pasteur pipette was used to agitate tissue chunks for further digestion. The cell solution was filtered through open mesh fabric with a pore size of 255 μm into a 50 ml conical tube. After the isolated cells had settled, the supernatant was removed and the cell pellet was washed twice with a room‐temperature solution of 1 mg/ml BSA in isolation buffer. [Ca^2+^] was gradually increased to the desired amount, starting from 0.1 mmol/l Ca^2+^.

### Scanning electron microscopy

Eight millimetre round glass coverslips (Electron Microscopy Sciences, Hatfield, PA, USA) were autoclaved then coated with 0.01% poly‐l‐lysine (Sigma‐Aldrich) for 15 min at room temperature (RT), followed by 10 μg/ml natural mouse laminin (Thermo Fisher Scientific, Waltham, MA, USA) in Medium 199 (Sigma‐Aldrich) overnight at 4°C. Isolated cardiomyocytes were seeded on coated coverslips for 30–45 min then fixed in solution containing 2.5% glutaraldehyde/4% paraformaldehyde in isolation buffer (see ‘Cell isolation’) containing 100 mmol/l Hepes for 10 min at RT. After initial incubation, fixative was replenished and samples were maintained in the same fixative solution at 4°C for various amounts of time. Fixed samples were washed with dH_2_O three times then dehydrated with a graded series of ethanol washes (70%, 90%, 96%, 100% × 4). All remaining ethanol was removed by critical point drying in a Bal‐Tec CPD 030 Critical Point Dryer (Bal‐Tec AG, Balzers, Liechtenstein). Dried samples were mounted on metal stubs with conductive carbon adhesive pads and sputter coated with approximately 6 nm of platinum in a Cressington 308UHR Sputter Coater (Cressington Scientific Instruments UK, Watford, UK). Images were acquired on a Hitachi S‐4800 field emission scanning electron microscope (Hitachi Ltd, Tokyo, Japan).

The proportion of Z‐groove per Z‐spine was calculated by measuring the length of elevated membrane crests, which define each groove, and then dividing this value by the total length of the Z‐spine extending from the base of the groove. The proportion of Z‐spines with t‐tubules was obtained by dividing the number of Z‐spines with a visible t‐tubule lumen by the total number of Z‐spines analysed per cell.

### Confocal imaging of t‐tubules

Isolated cardiomyocytes were incubated with 25 μmol/l di‐8‐ANEPPS (Thermo Fisher Scientific) for 25 min at RT. After 15 min of labelling, cardiomyocytes were transferred to a perfusion chamber containing a 30 mm coverslip coated in 10 μg/ml natural mouse laminin (Thermo Fisher Scientific) in Medium 199 (Sigma‐Aldrich). Following dye wash‐out, the t‐tubule network was visualized on a Zeiss LSM710 (Carl Zeiss AG, Oberkochen, Germany) scanning confocal microscope equipped with a C‐Apochromat ×40/1.2 water immersion lens. Pixel sizes ranged from 0.148 to 0.259 μm with a dwell time of 12.6 μs per pixel. Optical sections had a Z‐width of 0.4 μm.

T‐tubule density and organization was measured with a custom image processing macro. In short, excess background was removed (rolling ball = 15 pixels) prior to rotating the image so that the long axis of the cell was horizontal. Contrast was enhanced to 0.8% saturated pixels before application of Mexican hat (radius = 3) and median (radius = 1) filters. Following segmentation via the Otsu algorithm (Otsu, 1979), t‐tubule density was measured by tracing a region of interest just inside the surface sarcolemma. Images were skeletonized before measuring t‐tubule organization via a custom macro.

### Rapid 2D confocal scanning of Ca^2+^ release and action potentials

Cells were seeded on laminin pre‐coated 30 mm coverslips, as described above. Attached cells were loaded with 20 μmol/l fluo‐4 AM (Thermo Fisher Scientific) and 1.8 μmol/l FM 1‐43FX (Thermo Fisher Scientific) for 10 min at RT for simultaneous assessment of Ca^2+^ release and t‐tubule localization. Dye was washed out and subsequent experiments were performed with superfusion of Hepes‐Tyrode buffer containing (in mmol/l): 140 NaCl, 5 Hepes, 0.5 MgCl_2_, 5.4 KCl, 0.4 NaH_2_PO_4_, 5.5 glucose, 1.0 CaCl_2_. Cardiomyocytes were field stimulated at 1 Hz and all recordings were taken on an LSM 7Live scanning confocal microscope (Zeiss) equipped with a C‐Apochromat ×40/1.20 water‐immersion lens. Fluo‐4 and FM 1‐43FX were excited at 488 nm and resulting signals were passed through a long‐pass 505 nm filter. Small intracellular areas of the Fluo‐4/FM 1‐43FX loaded cells were scanned every 1.5 ms, with pixel size and dwell time set at 160 nm and 17.1 μs, respectively. Each optical slice had a thickness of 1.7 μm. Local Ca^2+^ transients were analysed within small, 1 × 1 μm sections of the recordings, centred on transverse or longitudinal t‐tubules. Comparison was made with Ca^2+^ release at ‘orphaned’ RyR sites in developing and failing cells, positioned at irregular gaps between transversely oriented t‐tubules. Transient timing was calculated by measuring the period from the start of Ca^2+^ rise within the 1 × 1 μm section to the intensity at half‐maximal fluorescence (F_50_) measured for the overall transient.

Rapid 2D confocal scanning was similarly employed to examine electrical activation of t‐tubules during the action potential. Cells were loaded with 1× FluoVolt/PowerLoad (Thermo Fisher Scientific) for 20 min at RT, before wash‐out and superfusion with Hepes‐Tyrode buffer. Microscope settings were as described above for Ca^2+^ imaging, but with a broader scan area encompassing the entire cell scanned every 10 ms. The percentage of depolarized t‐tubules was calculated by first segmenting the t‐tubule network as described in ‘Confocal imaging of t‐tubules’. Pixels corresponding to the location of t‐tubules were then isolated by subtracting an inverted image of the t‐tubule skeleton from the three frames prior to cell contraction. The resulting image stack was then divided by the first frame in the stack, and FluoVolt signals above background were segmented using the Otsu algorithm (Otsu, 1979). The skeletonized t‐tubule network was then stitched onto the end of the thresholded FluoVolt stack, and a custom dyssynchrony macro was used to measure the fraction of t‐tubule pixels whose FluoVolt signal was above threshold.

### Immunocytochemistry and colocalization analysis

Eight‐well LAB‐TEK® chambered coverglasses (Thermo Fisher Scientific) were coated with poly‐l‐lysine (Sigma‐Aldrich) and laminin (Thermo Fisher Scientific) as described in ‘Scanning electron microscopy’. Isolated cardiomyocytes were seeded on a coated coverglass for 30–45 min prior to fixation with 4% paraformaldehyde in 100 mmol/l Hepes isolation buffer (see ‘Cell isolation’) for 5 min at RT. Cells were subsequently washed with phosphate‐buffered saline (PBS) and fixation was quenched with 100 mmol/l glycine in PBS. Membranes were permeabilized with 0.5% Triton X‐100 (Sigma) in PBS and non‐specific antigens were blocked by incubating in a solution containing 15.1 mmol/l NaCl, 1.75 mmol/l Na_3_C_6_H_5_O_7_, 5% normal goat serum, 3% BSA and 0.02% NaN_3_ for 2 h at RT. Primary and secondary antibodies were diluted in a solution containing 15.1 mmol/l NaCl, 1.75 mmol/l Na_3_C_6_H_5_O_7_, 2% normal goat serum, 1% BSA and 0.02% NaN_3_ and applied to cells over‐night at 4°C and for 2 h at RT, respectively. Cells were washed with PBS between most steps following fixation, but not prior to incubation with primary antibody. Finally, samples were mounted in ProLong® Diamond Antifade with 4′,6‐diamidino‐2‐phenylindole (DAPI; Thermo Fisher Scientific) and allowed to cure for 24 h at RT. The following antibodies were used: α‐actinin (Abcam, Cambridge, UK; cat. no. ab9465, RRID:AB_307264, 1:100), cacna1c (FP1, generously provided by Olivia R. Buonarati and Prof. J. Hell, UC Davis, USA), caveolin‐3 (Abcam cat. no. ab2912, RRID:AB_2291095, 1:100), RyR1 (Thermo Fisher Scientific cat. no. MA3‐916, RRID:AB_2183054, 1:100), F(ab′)_2_‐goat anti‐mouse IgG (H+L) secondary antibody, Alexa Fluor 488 (Thermo Fisher cat. no. A‐11017, RRID:AB_2534084, 1:200), F(ab′)_2_‐goat anti‐rabbit IgG (H+L) secondary antibody, Alexa Fluor 546 (Thermo Fisher Scientific cat. no. A‐11071, RRID:AB_2534115, 1:200).

All images were captured on an LSM‐800 scanning confocal microscope (Zeiss) equipped with a Plan‐Apochromat ×63/1.4 oil immersion lens and Airyscan (Zeiss) detector. Excitation and emission wavelengths of 493/517 and 557/572 were used for AF488 and AF546 fluorophores, respectively. Pixel size and pixel dwell time for 15‐day‐old and adult cardiomyocytes were 34 nm^2^/3.66 μs and 71 nm^2^/3.54 μs, respectively. Airyscan post‐processing in Zen 2.1 (Blue Edition; Zeiss) was applied to each image prior to export and subsequent analysis.

For analysis, LTCC and RyR channels were separated and background was removed (rolling ball = 20 pixels). A region of interest was traced encompassing the cell's interior and all extracellular signal was cleared from the image. Values for fractional overlap (Mander's correlation coefficient; MCC) were calculated in JACoP, and the resulting automatically generated thresholds were used when calculating overall protein density within the cell in Fiji. For orientation‐specific analysis, the RyR channel was processed, binarized then skeletonized prior to being separated into transverse and longitudinal elements via a custom macro. The transverse skeleton was then subjected to both a vertical and horizontal convolution filter (kernel sizes 17 × 17 and 13 × 13, respectively) and binarized. The resultant transverse mask was then used to isolate the fluorescence signal in either the transverse or the longitudinal direction of processed Airyscan images, and MCCs were calculated as described above. The density of intact dyads in each cell was calculated as follows:

Dyadic  density = LTCC  density % area × MC C Fraction  LTCC  containing  RyR

To measure RyR and LTCC density, Airyscan micrographs were processed as described above then automatically segmented using the default algorithm in Fiji (Schindelin *et al*. 2012). The area occupied by any visible nuclei was excluded when measuring RyR and LTCC density.

### Western blotting

Protein quantification was performed as described previously (Strand *et al*. 2013). In short, 30 mg of snap‐frozen, left ventricular tissue was homogenized in protein lysis buffer using a TissueLyserII (Qiagen, Hilden, Germany). Homogenates were spun down at 1500*g* for 10 min at 4°C, and the resultant supernatant was transferred to a new tube and stored at −70°C. Total protein samples were run on Criterion^TM^ TGX^TM^ pre‐cast gels, and then transferred to 0.45 μm polyvinylidene difluoride membranes (both from Bio‐Rad Laboratories, Inc., Hercules, CA, USA). Blots were blocked in non‐fat dairy milk (Sigma‐Aldrich) or casein (Roche Diagnostics, Oslo, Norway) in tris‐buffered saline‐tween buffer (TBS‐T) and incubated overnight with primary antibody at 4°C, followed by species‐specific horseradish peroxidase‐conjugated secondary antibody for 1 h at room temperature. Blots were developed with ECL prime (Amersham/GE Healthcare, Little Chalfont, UK) and visualized on an LAS‐4000 Luminescent Image Analyzer (Fujifilm, Tokyo, Japan). Membranes were stripped for re‐probing using Restore Western Blot stripping buffer (Thermo Fisher Scientific). Image processing and protein signal quantification were performed in ImageQuant TL (v2003.03; RRID:SCR_014246) and ImageJ (Schneider *et al*. 2012; RRID:SCR_003070), respectively.

Primary antibodies used were: amphiphysin II (Bin1; Santa Cruz Biotechnology (Dallas, TX, USA; cat. no. sc‐23918, RRID:AB_667901, 1:500), caveolin‐3 (Abcam, ab2912, RRID:AB_2291095, 1:2500), junctophilin‐2 (Jph2; Santa Cruz Biotechnology cat. no. sc‐51313, RRID:AB_2296391, 1:2500), vinculin (Sigma‐Aldrich cat. no. V9131, RRID:AB_477629, 1:100,000).

### Image analysis and statistics

All image processing and analysis was conducted in Fiji (Schindelin *et al*. 2012; RRID:SCR_002285) and all statistical analyses were performed in SigmaPlot 12.5 (Systat Software, Inc., San Jose, CA, USA; RRID:SCR_003210). Data are presented as means ± SEM. Comparison between two groups was performed by Student's two‐tailed *t* test. Multiple groups were compared using one‐way ANOVA with the Tukey *post hoc* test. Differences with a *P*‐value < 0.05 were deemed significant. With the exception of Figs 4 *E* and 9, the ‘*n*’ used for statistical tests relates to the number of analysed cells. In Fig. 4 *E* the ‘*n*’ relates to number of hearts, while in Fig. 9 it denotes the number of t‐tubule or orphaned RyR sites analysed.

## Results

### Cardiomyocyte surface topography remodelling during development and HF

**Figure 1:**
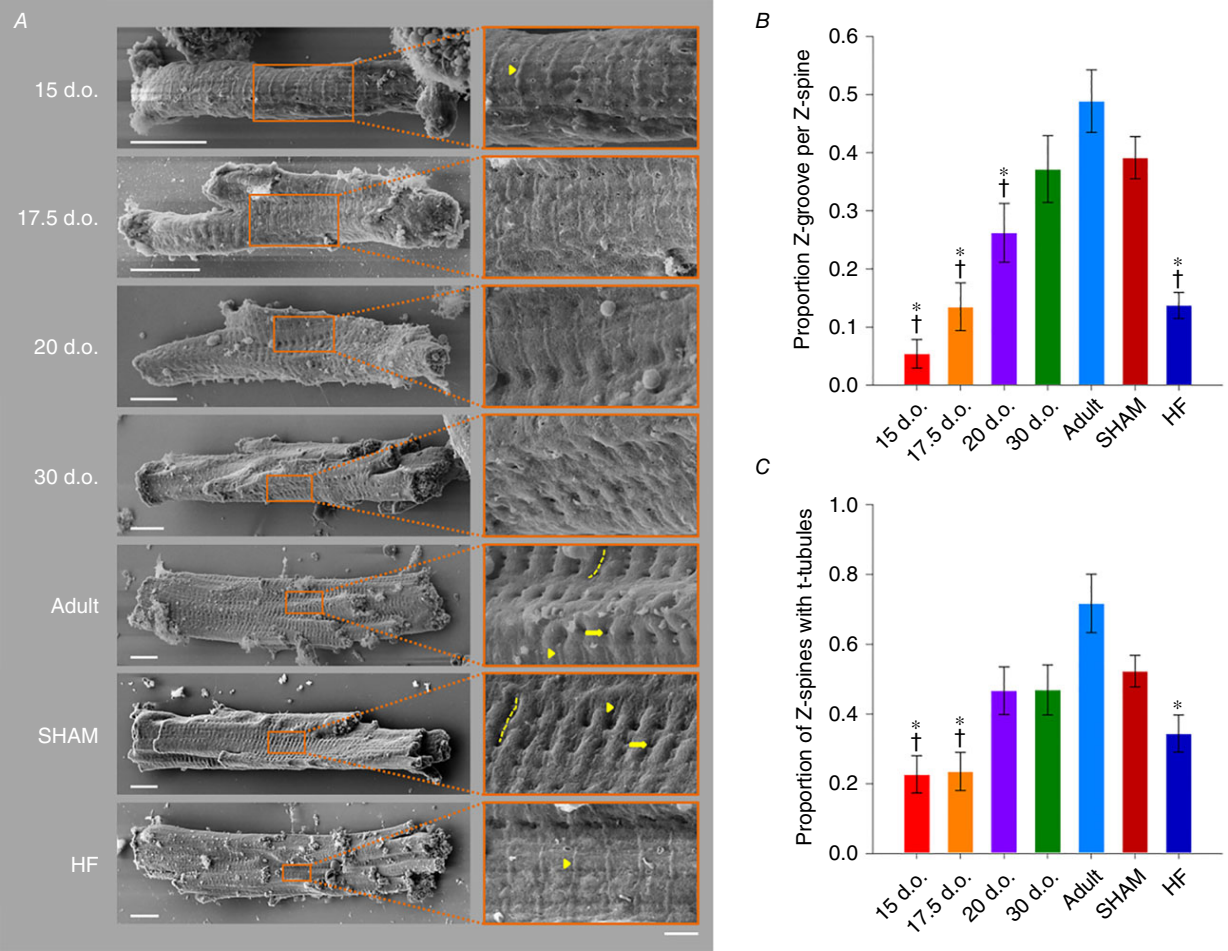
Remodelling of cardiomyocyte surface topography during development and heart failure *A*, representative scanning electron micrographs of isolated cardiomyocytes, with enlargements shown at right. Z‐grooves (dashed lines) and t‐tubule openings (arrows) appeared gradually during development, and were lost during HF. However, Z‐spines (arrowheads), corresponding to points of attachment of the surface membrane to the Z‐disk, were readily apparent from early developmental stages and in HF. In healthy adult cardiomyocytes, these Z‐spines were localized along the base of Z‐grooves (scale bars for left panels: 10 μm, for right panels: 2 μm). *B*, quantification of Z‐groove presence relative to Z‐spines. *C*, proportion of the cell with visible t‐tubule openings along Z‐spines. *n* = 18, 17, 15, 16, 15, 33, 32 cells from 3, 3, 3, 3, 3, 3, 3 hearts in 15 d.o., 17.5 d.o., 20 d.o., 30 d.o., adult, SHAM and HF. ^*^ *P* < 0.05 *vs*. adult, †*P* < 0.05 *vs*. SHAM calculated with one‐way ANOVA with a *post hoc* Tukey test. d.o., days old.

**Figure 2:**
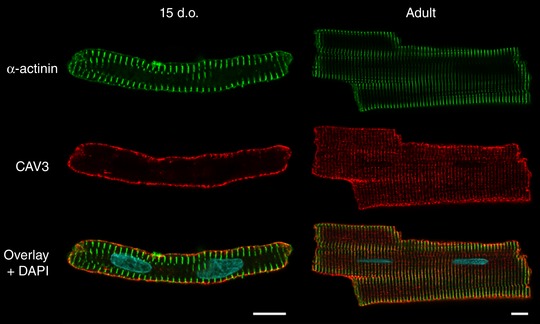
Z‐disks are arranged during early stages of development Representative confocal (Airyscan) images of immunolabelled cardiomyocytes show that the Z‐disk (α‐actinin staining) is present already at 15 days after birth, before the appearance of an organized t‐tubule network (caveolin‐3 staining). This observation supports the view that ‘Z‐spines’ observed by scanning electron microscopy at early stages of development (Fig. 1) likely result from points of attachment of the surface membrane with the Z‐disk. Scale bars: 10 μm. d.o., days old.

We employed the unparalleled resolving power of scanning electron microscopy to investigate changes in cardiomyocyte surface topography during postnatal development, adulthood and heart failure. Figure 1 *A* shows representative micrographs of isolated rat ventricular cardiomyocytes, with structural components of the cell surface highlighted in the enlarged panels. In agreement with previous descriptions, adult cardiomyocytes were observed to have an undulating, corrugated surface punctuated by regular ‘Z‐grooves’ (denoted by dashed lines), formed by elevated membrane crests on both sides of an underlying Z‐disc (Gorelik *et al*. 2006; Lyon *et al*. 2009). In adult cardiomyocytes, openings of t‐tubules from the base of these grooves were present at regular intervals (arrows). Neither Z‐grooves nor t‐tubule openings were apparent during the early postnatal period. However, the cell surface was not completely flat during early development, but rather exhibited regularly spaced elevated ridges (see arrowhead in Fig. 1 *A*, top panel), features that previous work has suggested may be attributed to sarcomeric structures (Sommer & Waugh, 1976; Semb *et al*. 1998). Indeed, we observed that Z‐grooves form gradually around these ridges during development, and that they remain present at the base of Z‐grooves even in mature cardiomyocytes. This suggests that the ridges, which we refer to as ‘Z‐spines’, result from subsarcolemmal attachment of the Z‐disk. Immunostains of α‐actinin and caveolin‐3 (Cav3) confirmed that the Z‐disks are laid down from early stages of development, prior to the delayed appearance of t‐tubules (Fig. 2). Due to their contiguous and robust appearance throughout development, we employed Z‐spines as a basis for quantification of the abundance of both Z‐grooves and t‐tubule openings, analyses which confirmed the gradual postnatal appearance of these features (Fig. 1 *B* and *C*).

**Figure 3:**
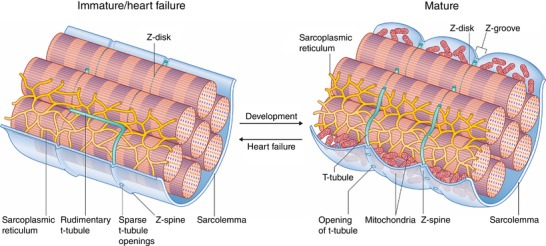
Summary of structural alterations to the surface and interior of cardiomyocytes during maturation and heart failure progression Points of attachment of the Z‐disk to the surface membrane create ridges referred to as ‘Z‐spines’, which are present from early stages of development. Upon maturation, Z‐grooves are formed as the surface membrane adjacent to each Z‐spine bulges outward. Previous work has suggested that these membrane crests are created by the presence of mitochondria (Poche, 1969; Kaprielian *et al*. 2000; Pasek *et al*. 2008). T‐tubules also appear gradually during development. These are initially apparent at the cell surface as sparse openings along Z‐spines, with a rudimentary internal structure that is largely longitudinal in orientation. With further maturation, the t‐tubules assume a denser and more transverse arrangement. During heart failure, both surface and internal structure revert to an immature phenotype. This remodelling includes loss of the dense, transversely oriented t‐tubule structure and flattening of the cell surface as Z‐grooves disappear.

Next, we investigated whether programmed remodelling of cardiomyocyte surface topography occurs during heart failure (HF). Myocytes isolated from HF rats 6 weeks following myocardial infarction were compared with sham‐operated controls (SHAM). We observed marked loss of Z‐grooves at the surface of failing cardiomyocytes, resulting in a smoother external membrane with easily visible Z‐spines (Fig. 1 *A* and *B*). Interestingly, this surface topography of failing cells was very similar to that of immature cardiomyocytes described above, as the measured proportion of Z‐grooves was comparable to that of cells isolated 17.5 days after birth (Fig. 1 *B*). T‐tubule openings within Z‐grooves were also less apparent in failing cells, in resemblance of the surface of developing cells (Fig. 1 *A* and *C*). These findings support that external cardiomyocyte topography develops gradually in the postnatal period and is dismantled as HF progresses. This paradigm is illustrated schematically in Fig. 3.

### Alterations in t‐tubule density and organization during development and disease

**Figure 4:**
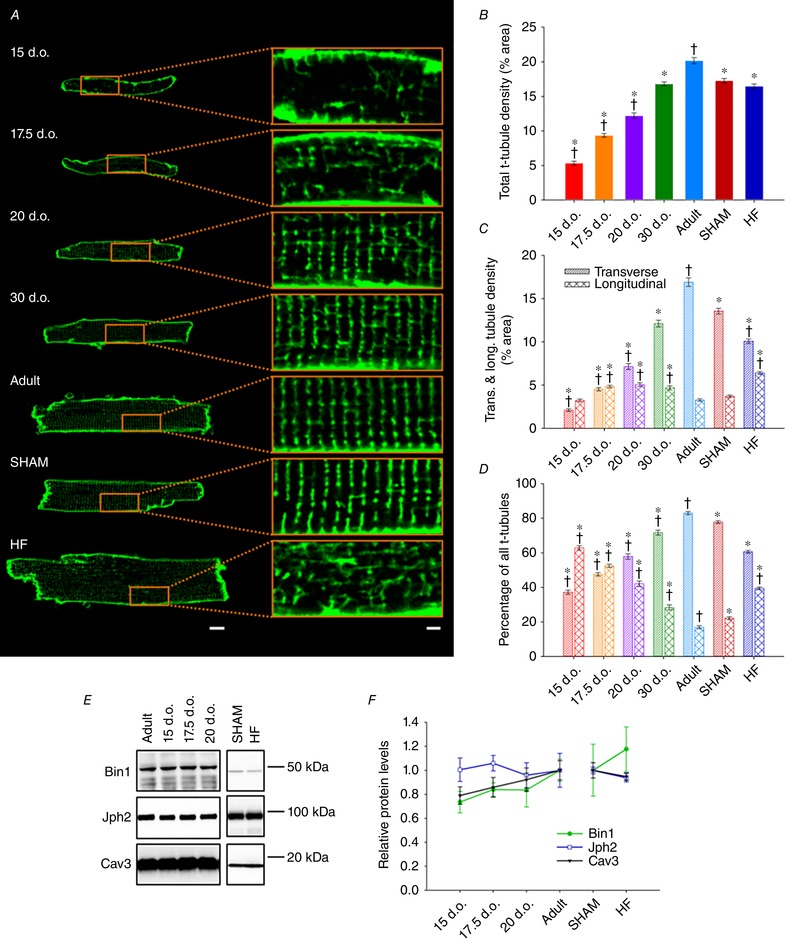
Spatial similarities between immature and pathologically remodelled t‐tubule networks *A*, representative confocal micrographs of isolated cardiomyocytes stained with di‐8‐ANEPPS for viewing t‐tubules, with enlargements shown at right (scale bars for left panels: 10 μm, for right panels: 2 μm). *B*, quantification revealed progressively increasing t‐tubule density during maturation, but no difference in t‐tubule density between SHAM and HF groups. *C*, longitudinal t‐tubules comprise the majority of early t‐tubule networks and are gradually replaced by transverse elements. *D*, HF is associated with re‐emergence of longitudinal t‐tubules while transverse elements are lost, producing a t‐tubule network reminiscent of immature cardiomyocytes. *n* = 62, 79, 56, 52, 72, 121, 166 cells from 4, 5, 4, 3, 3, 3, 4 hearts in 15 d.o., 17.5 d.o., 20 d.o., 30 d.o., adult, SHAM and HF. *E*, representative western blot portraying the levels of Bin1, Jph2 and Cav3 during development and HF. *F*, key regulators of t‐tubule growth and dyadic integrity were not significantly altered during either development or HF. For developmental time points (all proteins), *n* = 5, 5, 5, 6 hearts for 15 d.o., 17.5 d.o., 20 d.o. and adult. *n* (Bin1) = 6 and 6, *n* (Cav3) = 5 and 6, *n* (Jph2) = 4 and 4 hearts for SHAM and HF. ^*^ *P* < 0.05 *vs*. adult in same category, †*P* < 0.05 *vs*. SHAM in same category. All groups were compared by one‐way ANOVA with a *post hoc* Tukey test, with the exception of SHAM and HF in (*E*), which were compared via two‐tailed *t* test. d.o., days old.

We next investigated whether changing surface morphology during development and HF progression was mirrored at internal sites. T‐tubule density and organization were assessed by confocal microscopy in cardiomyocytes stained with di‐8‐ANEPPS (Fig. 4). T‐tubules were apparent from ∼15 days after birth, and appeared initially as small membrane invaginations that were often oriented longitudinally to the long axis of the cell. Since t‐tubule openings were not readily apparent at the cell surface at this time point (Fig. 1), the t‐tubule lumen may not yet be fully formed at these early stages. During maturation, overall t‐tubule density was observed to progressively increase (Fig. 4 *B*), and initial longitudinal elements were replaced by transverse t‐tubules (Fig. 4 *C* and *D*). Indeed, we found that longitudinal t‐tubules were largely transient in nature, reaching peak levels between 17.5 to 30 days after birth before decreasing in density as the cell reached adulthood.

While pathological remodelling of t‐tubules during the progression to HF is a well‐documented phenomenon (for review, see Louch *et al*. 2010; Guo *et al*. 2013; Manfra *et al*. 2017), we observed here that this structural reorganization shows reversion to an immature phenotype. During HF, t‐tubule changes specifically include loss of transverse elements and an increasing density of longitudinal elements, features which are robustly present in developing cells (Fig. 4). Indeed, the proportion of transverse and longitudinal t‐tubules was not significantly different between failing and 20‐day‐old cardiomyocytes (Fig. 4 *D*). Thus, as with external membrane structures, t‐tubules are restructured during HF, resulting in a remodelled network reminiscent of that present in immature cells (Fig. 3). Of note, at the time points of development and HF examined, we did not observe any change in the expression of the known dyadic regulators Bin1, Jph2, or Cav3 relative to adult or SHAM hearts (Fig. 4 *E* and *F*).

### Assembly and disassembly of Ca^2+^ handling proteins during development and HF

**Figure 5:**
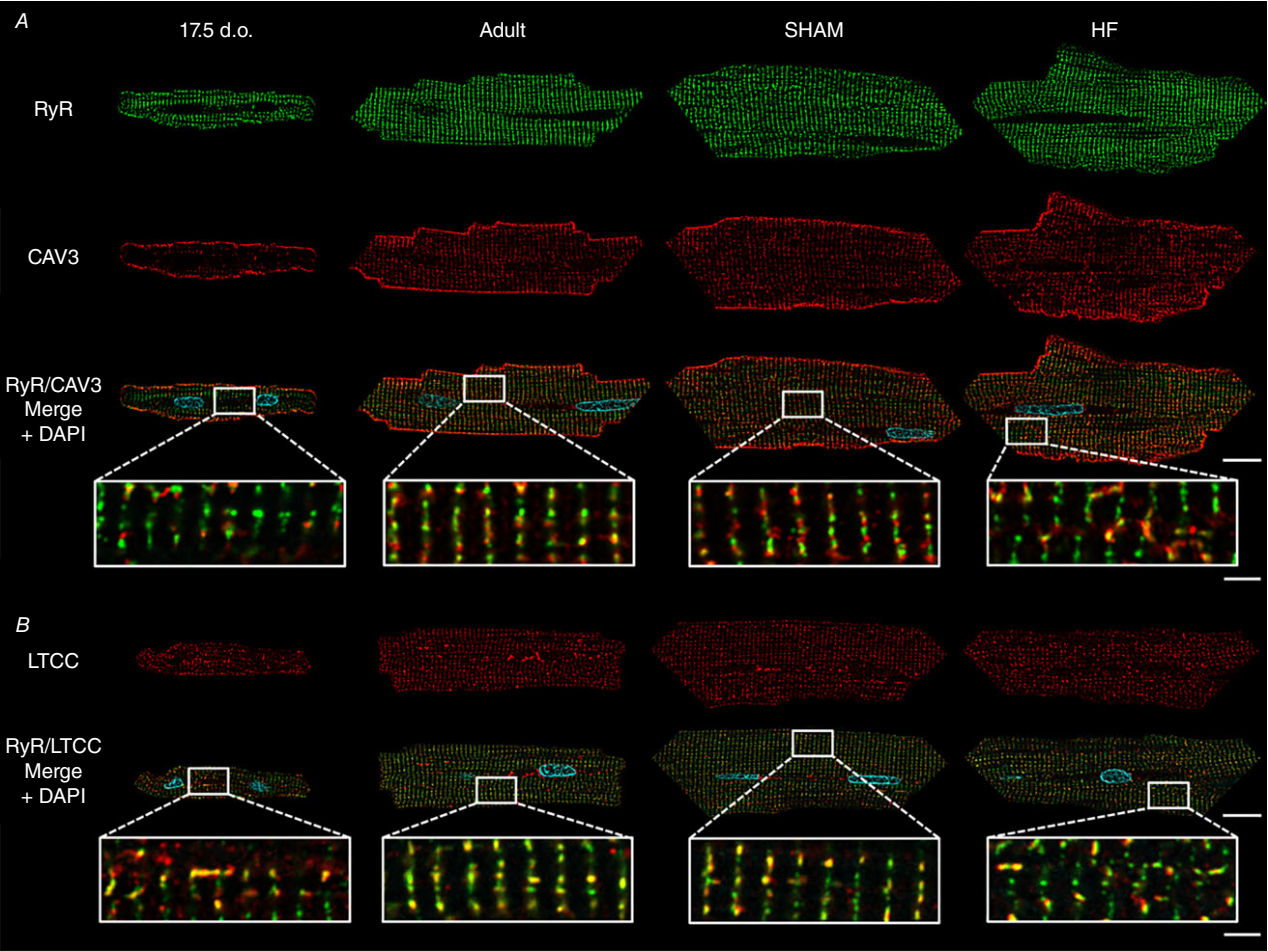
Representative super‐resolution confocal micrographs illustrating organization of cardiac dyads during development, adulthood and heart failure Isolated cardiomyocytes were immunolabelled with antibodies against RyRs in combination with either caveolin‐3 (*A*), to label t‐tubules, or LTCCs (*B*) and imaged using Airyscan super‐resolution confocal microscopy. A sparse network of ‘orphaned’ RyRs was observed to precede the arrival of t‐tubules and LTCCs along Z‐lines in developing cells. These orphaned RyRs reappeared during HF. However, where present, LTCCs were observed to be highly colocalized with RyRs, particularly along Z‐lines. Scale bars for left panels: 10 μm, for right panels: 2 μm. d.o. = days old.

**Figure 6:**
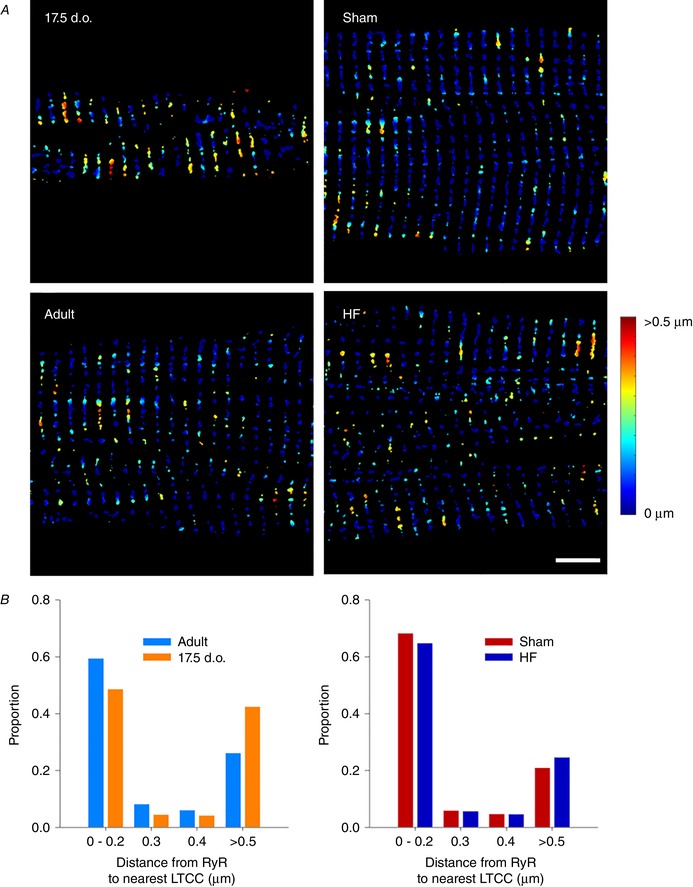
Calculation of RyR–LTCC distances *A*, thresholded Airyscan images were employed to calculate distance from RyR positions to nearest LTCCs, as indicated by the colour code (scale bar: 5 μm). *B*, in comparison with adult cardiomyocytes, developing cells exhibited a significantly right‐shifted distribution, consistent with a greater fraction of orphaned RyRs (*P* < 0.05 by *t* test). A similar tendency was observed in failing cells compared to Sham‐operated controls. *n* = 15, 15, 15, 15 cells from 3, 3, 3, 3 hearts in 17.5 d.o., adult, Sham and HF.

**Figure 7:**
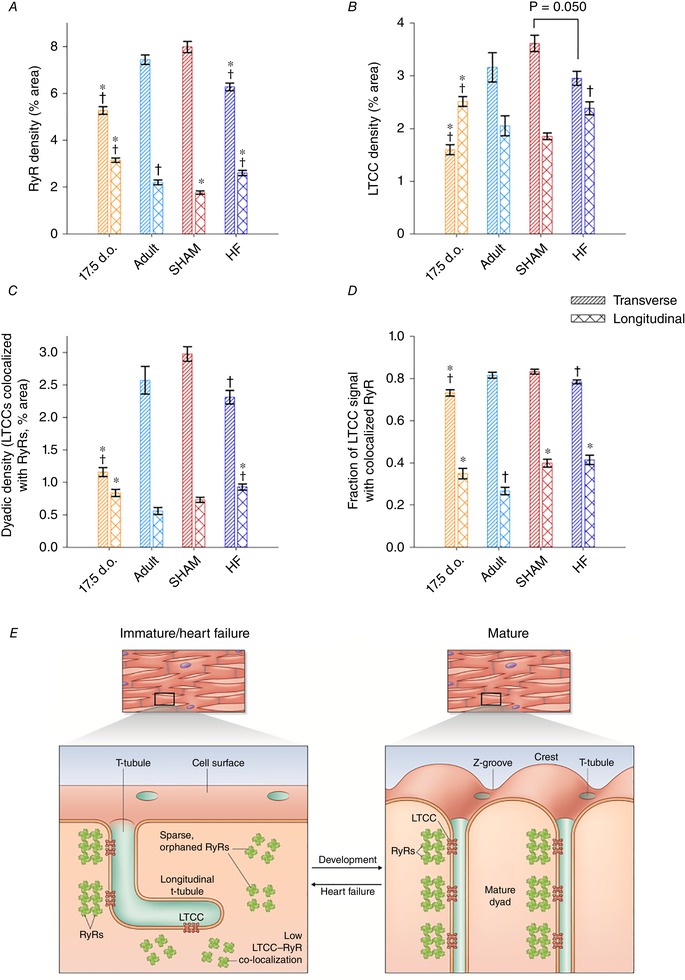
Packing and unpacking of dyads during development and heart failure *A*, RyR networks undergo considerable remodelling during development, as transverse elements replace longitudinal clusters. Conversely, transverse RyR clusters are lost while longitudinal clusters are gained during HF. *B*, a similar pattern of LTCC localization was observed, mirroring changes in t‐tubule morphology during development and HF (Fig. 4). *C*, colocalization analyses confirmed an increasing density of transversely localized dyads during development which is reversed during HF. *D*, the *proportion* of LTCCs colocalized with RyRs was always high along Z‐lines (transverse elements), indicating coordinated packing of these proteins into dyads during development and unpacking during HF. Of note, lower LTCC–RyR colocalization was observed along longitudinal t‐tubules of all cell types, suggesting that these structures have an immature dyadic makeup*. E*, observed similarities in cardiomyocyte substructure during development and disease are summarized in the schematic representaions. *n* = 44, 40, 39, 37 cells from 3, 3, 3, 3 hearts in 17.5 d.o., adult, SHAM and HF. ^*^ *P* < 0.05 *vs*. adult in same category, †*P* < 0.05 *vs*. SHAM in same category calculated with one‐way ANOVA with a *post hoc* Tukey test. d.o., days old.

To understand the functional implications of changes in t‐tubule structure in development, adulthood and HF, we examined the organization of t‐tubules and LTCCs relative to RyRs using super‐resolution AiryScan imaging. Representative images in Fig. 5 *A* and *B* show that, as expected, healthy adult cells exhibited a high density of transversely oriented t‐tubules (Cav3 staining) containing LTCCs, which were frequently colocalized with RyR clusters. Thus, distances measured from each RyR location to the nearest LTCC were generally short, and only a minority of RyRs could be clearly identified as being non‐junctional or ‘orphaned’ (Fig. 6 *A* and *B*). Quantification of the signal densities for these two proteins and colocalization analysis are illustrated in Fig. 7 *A–D*. Of note, we calculated that more than 80% of LTCCs located along transversely oriented tubules were colocalized with RyRs in healthy adult cells (Fig. 7 *D*). Although there are relatively few longitudinal tubules in healthy adult cells, these tubules were also observed to contain LTCCs and form dyadic junctions with RyRs. However, association between the two proteins was markedly lower along longitudinal elements than at their transversely oriented counterparts, with only a minority of longitudinal LTCCs observed to colocalize with RyRs (Fig. 7 *D*). This finding suggests that there is distinct organization of dyadic proteins along the two types of t‐tubules.

To address how these dyads are first assembled, we chose to examine 17.5‐day‐old cardiomyocytes as a developmental time point since they contain a nearly equal proportion of longitudinal and transverse t‐tubules. We observed that transversely aligned RyRs preceded the arrival of t‐tubules (Cav3 staining) and LTCCs (Fig. 5 *A* and *B*, respectively). Thus, measured distances from RyR locations to the nearest LTCCs were right‐shifted in comparison with mature cells, consistent with a greater proportion of orphaned RyRs (Fig. 6 *A* and *B*; *P* < 0.05 by *t* test). However, RyR clusters showed a more sparse distribution along Z‐lines at this stage than present in adult cardiomyocytes. Indeed, quantification revealed increasing RyR density with further maturation (Fig. 7 *A*). LTCC density in transverse t‐tubules similarly increased towards adulthood (Fig. 7 *B*), and colocalization analyses revealed an increasing density of transversely oriented dyads progressively packed with LTCCs and RyRs (Fig. 7 *C*). Of note, where present in developing cells, LTCCs already exhibited a high degree of colocalization with transversely oriented RyRs (Fig. 7 *D*), suggesting that these dyads may be functional even at early stages.

Mirroring t‐tubule morphology in developing cells, LTCC imaging revealed greater allocation to longitudinally oriented t‐tubules than is observed in mature cardiomyocytes (Figs 5 *B* and 7 *B*). While there was also a significant fraction of longitudinally oriented RyRs in these cells (Fig. 7 *A*), colocalization analysis showed that, as in adult cells, only a minority of LTCCs were associated with RyRs at these sites (Fig. 7 *D*). Thus, longitudinal tubules and dyads may be viewed as rather immature structures, which are prominent in developing cells but still found at lower density in healthy adult cells. The two dyadic arrangements are schematically illustrated in Fig. 7 *E*.

During HF progression, dyadic organization showed a reversion to an immature phenotype. In agreement with previous work, we observed that disorganization of t‐tubular structure appeared to result in the formation of orphaned RyRs at gaps between transverse elements (Fig. 5 *A* and *B*). Indeed, measurements of RyR distances to nearest LTCC tended to be right‐shifted in failing cells (Fig. 6 *A* and *B*), and significantly fewer dyadic junctions were observed (Fig. 7 *C*). However, the transversely oriented RyR network remained only partially in place in failing cells, as quantification revealed an overall loss of RyR staining which paralleled changes in LTCCs (Fig. 7 *A* and *B*). Importantly, where t‐tubules and LTCCs remained present, proportional colocalization with RyRs remained high (Fig. 7 *D*), indicating that loss of LTCCs and RyRs occurred predominantly at the same sites. Thus, the SR appears to reorganize in tandem with t‐tubule structure during HF, resulting in an *unpacking* of both RyRs and LTCCs from transversely oriented dyads. In further similarity to developing cells, an increased allotment of LTCCs and RyRs to newly formed longitudinal dyads was also observed in failing cells (Fig. 7 *A–C*). As in the other cell groups examined, LTCCs and RyRs exhibited only modest colocalization along longitudinal tubules in these cells (Fig. 7 *D*). These findings are summarized in Fig. 7 *E*.

### T‐tubules are functional from early developmental stages, but lose functionality during HF

**Figure 8:**
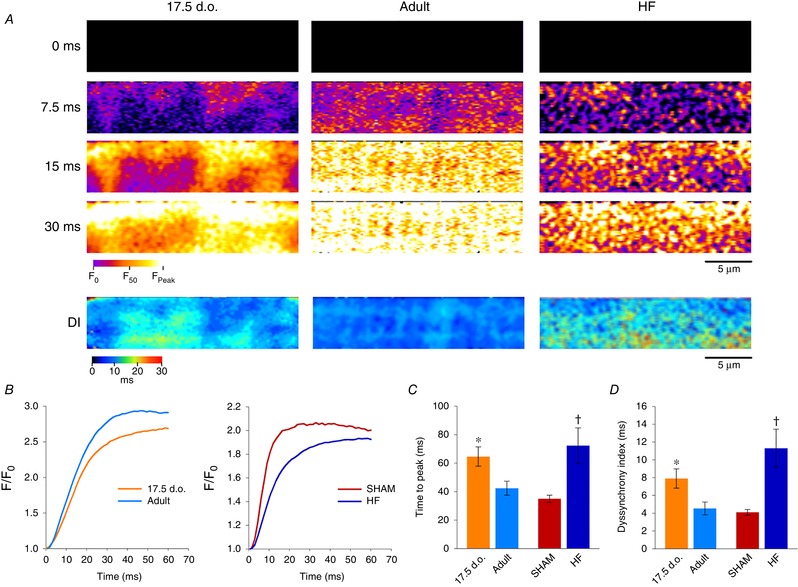
Immature and failing cardiomyocytes exhibit slow, dyssynchronous Ca^2+^ transients *A*, representative 2D confocal scans are presented from developing, adult and HF myocytes, at selected time frames during the early phase of the Ca^2+^ transient (fluo‐4 AM). Full videos are provided (Supporting information, Videos S1 and S2). More dyssynchronous Ca^2+^ release was observed in immature and failing cardiomyocytes, as highlighted by dyssynchrony index (DI) maps illustrating the time to half‐maximal fluorescence across the cell (lower panels). *B*, representative traces of Ca^2+^ transients from all groups. *C* and *D*, quantification of Ca^2+^ release kinetics confirmed that transients from immature and failing cardiomyocytes are both slower (*C*) and more dyssynchronous (*D*) than their respective controls. For time to peak: *n* = 18, 15, 13, 14 from 6, 3, 5, 5 hearts for 17.5 d.o., adult, SHAM and HF. For DI: *n* = 18, 15, 13, 11 from 6, 3, 5, 4 hearts for 17.5 d.o., adult, SHAM and HF. ^*^ *P* < 0.05 compared to adult; †*P* < 0.05 compared to SHAM. DI, dyssynchrony index; d.o., days old.

Having characterized changes to both sarcolemmal organization and key Ca^2+^ handling components during development and HF, we next investigated how these changes affect Ca^2+^ release in immature and failing cardiomyocytes. Ca^2+^ release was assessed by means of a 2D confocal time series of isolated cardiomyocytes loaded with Fluo‐4 AM. As expected based on their disorganized t‐tubule networks, both developing and HF cardiomyocytes were observed to produce slower, more dyssynchronous Ca^2+^ transients than their respective controls (Supporting information Video S1 and Fig. 8). Times to peak measurement were found to be significantly prolonged compared to healthy adult cells (Fig. 8 *C*) and Ca^2+^ release was more heterogeneous, as evidenced by dyssynchrony maps (Fig. 8 *A*, lower panels) and increased dyssynchrony indices (Fig. 8 *D*).

**Figure 9:**
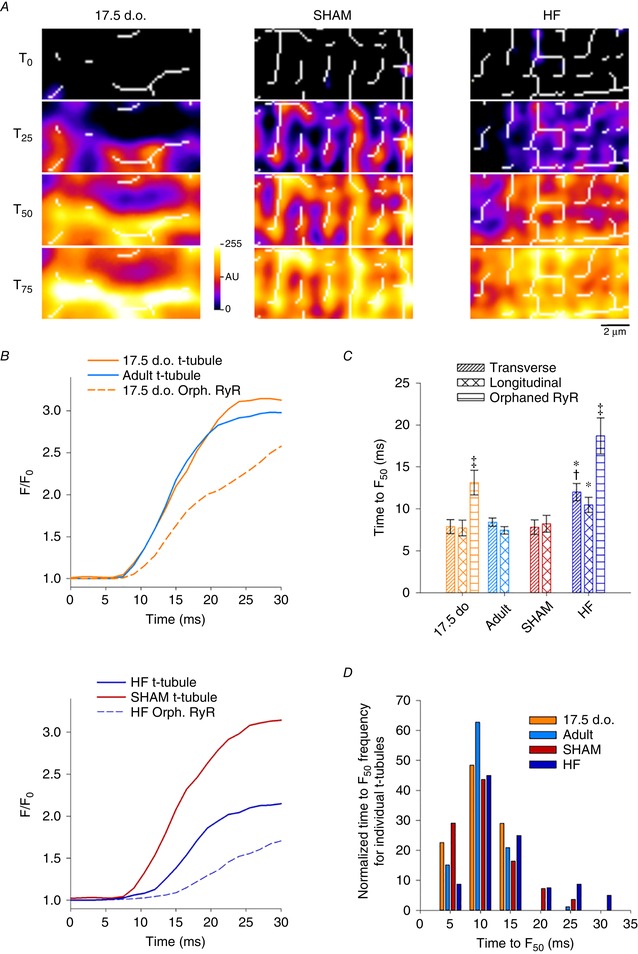
T‐tubules effectively trigger Ca^2+^ release in immature but not failing cardiomyocytes *A*, overlay of 2D Ca^2+^ release patterns and skeletonized t‐tubule networks from representative 2D confocal videos (as in Fig. 8). Local Ca^2+^ release was examined within small (1 × 1 μm) regions corresponding to intact transverse or longitudinal t‐tubules, or orphaned sites. *B*, representative local transients show rapid Ca^2+^ release along t‐tubules in developing and healthy adult cells, but slowed Ca^2+^ rise at t‐tubules in HF cardiomyocytes. As expected, orphaned RyR sites exhibited delayed Ca^2+^ release in both developing and failing cells, dependent on Ca^2+^ propagation from intact dyads. *C* and *D*, time to half‐maximal fluorescence (TTF_50_) measurements confirmed loss of t‐tubule functionality in HF. For the purpose of display, local transients were smoothed with a 5 point moving average to reduce noise. *n*_transverse_ = 17, 46, 31, 38; *n*_longitudinal_ = 14, 40, 24, 42; *n*_OrphRyR_ = 14, 0, 0, 33 from 4, 3, 3, 6 hearts in 17.5 d.o., adult, SHAM and HF. ^*^ *P* < 0.05 *vs*. adult in same category, †*P* < 0.05 *vs*. SHAM in same category, ‡*P* < 0.05 *vs*. transverse and longitudinal tubule in same group. Significance was calculated by one‐way ANOVA with a *post hoc* Tukey test. d.o., days old.

We further characterized the function of t‐tubules themselves by examining local Ca^2+^ release in 2D confocal scans. This was done in experiments with simultaneous labelling of t‐tubules (FM 1‐43FX) and Ca^2+^ (Fluo‐4), to enable assessment of local Ca^2+^ release within small (1 × 1 μm) regions of interest corresponding to t‐tubules or orphaned RyRs (Fig. 9 *A*). In healthy adult cells, Ca^2+^ release was initiated rapidly from RyRs located along both transverse and longitudinal t‐tubules, as indicated by representative videos (Supporting information, Video S2), selected time points (Fig. 9 *A*) and local Ca^2+^ transients (Fig. 9 *B*). Thus, although LTCC and RyR colocalization is lower at longitudinal compared to transverse elements (Fig. 7 *D*), both types of dyads can efficiently trigger Ca^2+^ release. At t‐tubule sites in developing cells, Ca^2+^ release was also efficiently triggered (Fig. 9 *B* and *C*). This finding indicates that the overall desynchronized and slowed Ca^2+^ transient observed in these cells results rather from the disorganized and low density of t‐tubules, which creates abnormal gaps between them. Indeed, local transients measured at these gaps showed delayed Ca^2+^ release (Fig. 9 *B* and *C*), in agreement with previous work showing that orphaned RyRs are triggered only after Ca^2+^ propagates from intact dyads (Louch *et al*. 2006; Song *et al*. 2006). In contrast, Ca^2+^ release was observed to be impaired at t‐tubular sites in failing cardiomyocytes (Fig. 9 *B* and *C*). This deficit was manifested as a markedly slower local Ca^2+^ transient, particularly at transverse elements where time to *F*_50_ values were significantly prolonged in comparison with equivalent regions in Sham (Fig. 9 *C*). Thus, slow and dyssynchronous Ca^2+^ transients observed in failing cardiomyocytes are attributed to both immature organization of t‐tubules *and* dyadic dysfunction.

**Figure 10:**
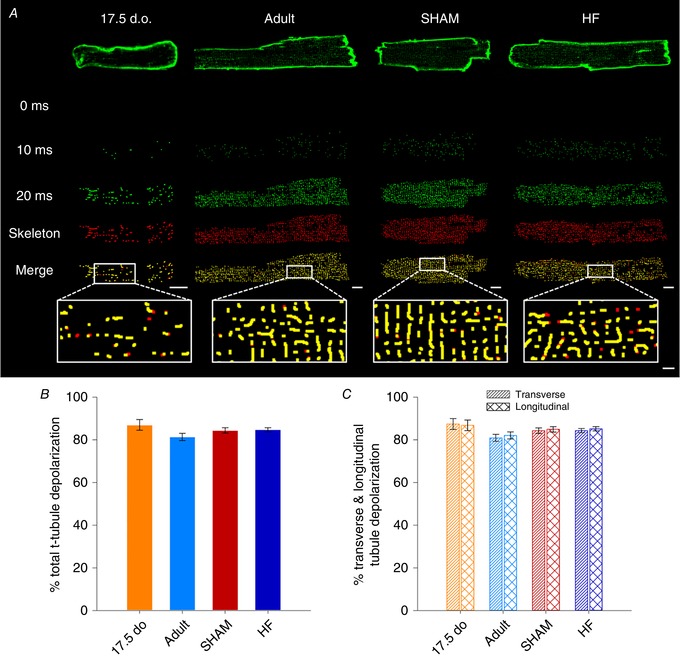
Action potential propagation is not deficient along immature or failing t‐tubule networks *A*, representative 2D confocal images of isolated cardiomyocytes loaded with FluoVolt, at selected time points following the onset of electrical stimulation. Shown below each times series is the cell's skeletonized t‐tubule network (red), and the proportion of t‐tubules for which fluorescence increased above threshold (Otsu) during the action potential (green). *B* and *C*, similar extents of t‐tubule depolarization were observed in developing, adult and HF cardiomyocytes. *n* = 15, 35, 54, 77 cells from 6, 3, 5, 4 hearts for 17.5 d.o., adult, SHAM and HF. Differences between groups were compared via one‐way ANOVA. d.o., days old.

Previous studies have suggested that t‐tubule malfunction can be caused by incomplete propagation of the action potential during HF (Crocini *et al*. 2014). In order to investigate this as a plausible mechanism, we loaded cardiomyocytes with a voltage‐sensitive dye (FluoVolt) and performed rapid 2D confocal scanning of cell‐wide t‐tubule networks (Fig. 10 *A*). Unlike previous reports, we did not observe any differences in the extent of action potential propagation in failing cardiomyocytes. As illustrated in Fig. 10 *B* and *C*, a similar fraction of the t‐tubule network was depolarized in failing, immature and control t‐tubule networks. Rather, the non‐functionality of t‐tubules appears to be consistent with reduced t‐tubular Ca^2+^ current, a phenomenon recently reported by others in failing cells (Bryant *et al*. 2015; Sanchez‐Alonso *et al*. 2016).

## Discussion

We here report striking parallels in cardiomyocyte substructure during cardiac development and heart failure. At the cell surface, we observed that Z‐grooves and t‐tubules appear gradually during cardiac development, and that these structures disappear during HF in resemblance to rather late postnatal periods. Similar parallels in t‐tubule and dyadic structure occur within the cell interior, with a ‘last in, first out’ pattern evident as the latest stages of dyadic development are reversed during disease. This pattern is evidenced by a disorganization of t‐tubules in failing cells, with fewer transverse elements and a high proportion of longitudinal tubules reminiscent of the late stages of postnatal development. At the dyadic level, we observed that RyRs are laid down in advance of developing t‐tubules and are similarly ‘orphaned’ in failing cells. However, nanoscale imaging revealed that the Z‐line distribution of RyRs is relatively sparse in these cells, indicating that there is coordinated packing and unpacking of these channels in dyadic junctions with LTCCs. The disorganized organization and lower density of dyads in immature and failing cells is critically linked to desynchronized and slowed Ca^2+^ release in these two states. However, while developing cells exhibit efficient triggering of Ca^2+^ release at newly formed dyads, dyadic function is impaired in failing cells despite similar organization of Ca^2+^ handling proteins. Thus, pathologically deficient Ca^2+^ homeostasis during heart failure is only partly linked to the re‐emergence of immature subcellular structure, and likely additionally reflects lost t‐tubule functionality.

### Plasticity of surface sarcolemmal organization

The surface sarcolemma of immature cardiomyocytes has a smooth appearance with regular transverse striations (Fig. 1). This unique surface characteristic has previously been described in both mouse and rat cardiac muscle, but earlier studies were unable to conclude whether these striations were the result of t‐tubules or other subsarcolemmal structures located at the Z‐disc (Sommer & Waugh, 1976; Semb *et al*. 1998). We propose that these striations, or Z‐spines as we refer to them, are sites of attachment between the surface sarcolemma and the developing myofibrils. Indeed, we observed that labelling of α‐actinin, an anchoring protein located at the Z‐disc, showed regular striations in 15‐day‐old cells, a time point when an extensive t‐tubule network is not yet present (Fig. 2). Previous work has shown that Z‐disc organization in fact begins *in utero*, with attachment sites between electron‐dense Z material and surface sarcolemma noted as early as 10 days post‐coitus (Markwald, 1973). Thereafter, myofibril development continues toward the cell interior as the cardiomyocyte matures (for review see Smolich, 1995; see also α‐actinin staining in Fig. 2). The fact that Z‐spines remain a prominent feature of the sarcolemma of failing cardiomyocytes supports our belief that disease progression includes reversal of relatively late stages of development, while Z‐disk formation and attachment are early processes initiated *in utero*.

Unlike, Z‐discs and Z‐spines, we observed here that Z‐grooves develop relatively late in the postnatal period (20–30 days of age) and are also readily lost during HF (Fig. 1 *B*). These structures have been described by Gorelik *et al*. (2006), using scanning ion conduction microscopy to obtain a high‐resolution surface image of small sections of cardiomyocytes. In their work, the Z‐groove abundance, or the Z‐index, was quantified by dividing the measured length of a visible Z‐groove by the total estimated Z‐groove length across the entire image frame. Our approach is similar; however, we made use of the unparalleled resolving power of scanning electron microscopy in order to image the cardiomyocyte surface in even greater detail. Our observation that Z‐grooves are highly malleable structures supports previous work, as prolonged culturing of cardiomyocytes, genetic manipulation and cardiac disease can cause their destruction (Gorelik *et al*. 2006; Lyon *et al*. 2009; Ibrahim *et al*. 2013). Similarly, mechanical unloading of cardiomyocytes is either beneficial or detrimental to surface topography depending on the setting under which it is applied (Ibrahim *et al*. 2010, 2012). It is not just a matter of aesthetics: remodelling of cardiomyocyte surface architecture can have profound effects on cell function. Using a HF model induced by coronary ligation, Lyon and colleagues found that loss of Z‐grooves was accompanied by partial redistribution of β_2_‐adrenergic receptors (β_2_‐ARs) to the membrane crest between adjacent Z‐grooves (Lyon *et al*. 2012). This mislocalization of β_2_‐ARs in failing cardiomyocytes resulted in a blunted contractile response when cells were stimulated with isoprenaline. Thus it appears that Z‐grooves are important structural components that help to regulate cardiomyocyte function, and that their loss during disease is pathological.

The mechanisms behind Z‐groove formation and destruction remain elusive; however, others have argued that they form in part due to translocation of mitochondria during cardiac development, as illustrated in Fig. 3. As cardiomyocytes mature, mitochondria increase in number and size and translocate from the cytoplasm to the perinuclear, interfibrillar and subsarcolemmal space (Olivetti *et al*. 1980; Canale *et al*. 1986; Ong & Hausenloy, 2010). Indeed, previous studies have shown that subsarcolemmal mitochondria can be found underneath membrane crests that span the length of individual sarcomeres, thereby creating valleys that overlay the Z‐disc (Poche, 1969; Kaprielian *et al*. 2000; Pasek *et al*. 2008). Mitochondria are adversely affected during HF, as electron micrographs of dilated failing human myocardium revealed large intracellular areas lacking myofibrils that contained clusters of small, fragmented mitochondria (Schaper *et al*. 1991). Therefore, HF‐induced fragmentation and rearrangement of mitochondria is one possible mechanism that could contribute to Z‐groove destruction.

### Plasticity of t‐tubules and dyads

T‐tubules are also extremely versatile structures capable of adapting to different developmental and pathological stimuli. Although species like cow (Forsgren & Thornell, 1981), guinea pig (Forbes & Sperelakis, 1976), sheep (Sheldon *et al*. 1976; Brook *et al*. 1983) and human (Kim *et al*. 1992) have cardiac t‐tubules from birth, they are not found in altricial species like mice (Ishikawa & Yamada, 1975; Hamaguchi *et al*. 2013), rabbit (Hoerter *et al*. 1981) or rat (Hirakow & Gotoh, 1975). By visualization of t‐tubule openings on the cell surface (Fig. 1) and confocal imaging of internal t‐tubules by confocal microscopy (Fig. 4), we have observed that t‐tubule density progressively increases after birth in rats. Although the initial orientation of developing t‐tubules is largely longitudinal, these elements are progressively replaced by a larger fraction of transverse elements as the cardiomyocyte reaches maturity. This process is readily reversed during HF, with loss of transverse elements and re‐emergence of longitudinal elements. While the similarities in t‐tubule structure between failing and immature cardiomyocytes have not been previously characterized in detail, a large body of work has described t‐tubule remodelling during HF (Louch *et al*. 2010; Guo *et al*. 2013; Manfra *et al*. 2017). Plasticity of t‐tubule structure is also supported by previous work examining effects of cardiac resynchronization therapy (Sachse *et al*. 2012), exercise training (Stolen *et al*. 2009; Kemi *et al*. 2011), gene therapy (Lyon *et al*. 2012) and mechanical unloading (Ibrahim *et al*. 2012). These findings support that remodelling of t‐tubules during both health and disease is a programmed response.

Beyond t‐tubule structure, our data provide additional insight into the composition of dyads during development, adulthood and disease. In agreement with previous work (Ziman *et al*. 2010), we observed that RyRs are laid down along Z‐lines in advance of the arrival of T‐tubules in developing cells. However, the Airyscan super‐resolution imaging employed here revealed that this early distribution of RyRs is sparse, and that RyRs continue to be packed into developing dyads along with LTCCs as t‐tubules grow (see schematic representation in Fig. 7 *E*). High colocalization of LTCCs with RyRs from early stages suggests that dyadic packing is a carefully controlled process in the developing heart, which allows t‐tubules to become functional shortly after they are grown (Fig. 9). Structurally, we observed that dyadic organization reverts to an immature phenotype during HF, as t‐tubule regression and dyadic unpacking occur simultaneously. This includes loss of LTCCs along with t‐tubules, but also loss of some RyRs along Z‐lines, resulting in a more sparse distribution (see also Kolstad *et al*. 2018). However, where they are still present, LTCCs remain localized in dyads, i.e. closely colocalized with RyRs (Fig. 7 *D*).

Of note, in all cell groups examined, the composition of longitudinally oriented dyads appears to be distinct from that of transverse elements, with lower colocalization of LTCCs with RyRs (Fig. 7 *D*). While this differential dyadic arrangement does not appear to dramatically alter the ability of longitudinal dyads to trigger Ca^2+^ release (Fig. 9), it does suggest that longitudinal tubules may have specialized roles that are well‐suited to the developing heart where they are prominent. For example, we previously observed high colocalization of the Na^+^–Ca^2+^ exchanger in longitudinal dyads with RyRs (Swift *et al*. 2012) and indeed, exchanger activity is known to be prominent in the developing heart (reviewed in Louch *et al*. 2015). The re‐emergence of longitudinal dyads in the failing heart, therefore, has important implications for understanding both Na^+^ and Ca^2+^ homeostasis in this condition.

The striking similarities in tubule/dyadic structure between developing and failing cardiomyocytes is strongly suggestive of underlying gene programmes which are shared in the two conditions. Included pathways may comprise both immature genes which are reactivated in the diseased heart, and adult genes which are suppressed (for review see Rajabi *et al*. 2007). While these programs have not been previously linked to t‐tubule and dyadic integrity, we hypothesized that their downstream targets may include established dyadic regulators such as Jph2 or Bin1. However, while both proteins have been shown to be critical for dyadic assembly in early development (Chen *et al*. 2013; Reynolds *et al*. 2013), we observed that by later stages their expression was not markedly different from the adult heart (Fig. 4 *F*). Furthermore, we did not observe marked changes in Jph2 or Bin1 expression in failing hearts, suggesting that loss of these proteins is not a critical initiator of dyadic remodelling. Nevertheless, we believe that with even greater severity of HF in some animal models and patients (Minamisawa *et al*. 2004; Wagner *et al*. 2012; Hong *et al*. 2012a,*b*), loss of these proteins may be triggered as the developmental phenotype is rolled back to even earlier stages. Indeed, in the rat post‐infarction HF model currently employed, we have observed that Jph2 loss occurs proximal to the infarct where dyadic remodelling is most severe (Frisk *et al*. 2016). While we have not performed such region‐specific analyses in the present study, an intriguing hypothesis is that gene programmes critical for dyadic maintenance may be activated or repressed on a local basis across the failing ventricle.

### Faulty t‐tubules contribute to slower, dyssynchronous Ca^2+^ release in failing cardiomyocytes

A major consequence of t‐tubule loss is disruption to cardiomyocyte Ca^2+^ release. We and others have previously shown that t‐tubule disruption, as a result of either prolonged culturing or pathological states, impairs Ca^2+^ release synchronicity in cardiomyocytes (Louch *et al*. 2004, 2006; Heinzel *et al*. 2008; Frisk *et al*. 2016). In this study we have used rapid confocal scanning to image live Ca^2+^ release from a 2D intracellular area within the cardiomyocyte. When comparing Ca^2+^ release kinetics in developing and failing cardiomyocytes, we see that both cell types produce Ca^2+^ transients that are slower and more dyssynchronous than their respective controls. Initial studies attributed slower, dyssynchronous Ca^2+^ release in failing cardiomyocytes to irregular gaps between adjacent t‐tubules and the delayed arrival of trigger Ca^2+^ to orphaned RyRs (Song *et al*. 2006). Recent evidence, however, has suggested that defective t‐tubules may also contribute to alterations in Ca^2+^ release during HF (Crocini *et al*. 2014; Bryant *et al*. 2015; Sanchez‐Alonso *et al*. 2016). Our results using simultaneous t‐tubule and Ca^2+^ imaging support this view, as Ca^2+^ release along t‐tubule networks in failing cells was indeed observed to be slower than in adults and SHAM‐operated controls (Fig. 9). Such impaired t‐tubule function was not shared by developing cells, despite similar t‐tubule organization and dyadic composition in the two states. A series of papers have indicated that L‐type Ca^2+^ current is lost from t‐tubules during HF, and redistributed to the surface membrane (Bryant *et al*. 2015, 2018; Sanchez‐Alonso *et al*. 2016). An underlying role of loss of t‐tubular Cav3 has been highlighted, which is proposed to reduce the stimulatory influence of this protein on Ca^2+^ current. Conversely to Bryant and colleagues, who employed a pressure‐overload mouse model of HF, we did not observe similar changes in Cav3 levels in failing rat hearts following myocardial infarction. Such a discrepancy could be attributed to species‐ or model‐specific differences in pathophysiology, and is likely dependent on the extent of HF progression. Additionally, our results appear not to be in agreement with an alternative theory put forward by Crocini and colleagues suggesting that t‐tubule dysfunction in heart failure is linked to deficient action potential propagation (Crocini *et al*. 2014).

In conclusion, our present findings indicate that disorganization of cardiomyocyte structure during heart failure reflects a reversion to an immature phenotype manifested at both the cell surface and the interior. This rearrangement appears to be carefully orchestrated, with preferential reversal of the latest stages of postnatal maturation. Thus, we expect that improved understanding of genetic and/or epigenetic similarities between the two states may provide new opportunities for preventing subcellular degradation and weakened cardiomyocyte contraction during HF progression. Importantly, however, our data indicate that dyadic dysfunction during HF also includes mechanisms distinct from the developing heart, further highlighting the complexity of this disease.

## Additional information

### Competing interests

None declared.

### Author contributions

D.B.L. and W.E.L. were responsible for the conception and design of the study. D.B.L., M.F., J.W.H. and O.R.B. contributed to cellular imaging and analysis, while animal surgery and imaging were performed by J.M.A., E.S.N. and I.S. Study supervision was performed by J.W.H., I.S., G.C. and W.E.L. All authors contributed to the writing and critical review of the manuscript. All authors have read and approved the final version of this manuscript and agree to be accountable for all aspects of the work in ensuring that questions related to the accuracy or integrity of any part of the work are appropriately investigated and resolved. All persons designated as authors qualify for authorship, and all those who qualify for authorship are listed.

### Funding

This work was supported by the European Union's Horizon 2020 research and innovation programme (Consolidator grant, WEL) under grant agreement No. 647714. Additional support was provided by The South‐Eastern Norway Regional Health Authority, Anders Jahre's Fund for the Promotion of Science, The Norwegian Institute of Public Health, Oslo University Hospital Ullevål and the University of Oslo.

## Supporting information

Video S1. 2D Confocal time‐lapse recordings of Ca2^+^ transients
Video S2. 2D Confocal time‐lapse recordings of Ca2^+^ transients and t‐tubules
