# Supplementary material for: Cardiomyocyte substructure reverts to an immature phenotype during heart failure
Source: J Physiol. 2019 Feb 27;597(7):1833–53. doi: 10.1113/JP277273 (PMC6441900; doi:10.1113/JP277273)
Supplement: Supplementary file 1 — Video S1. 2D Confocal time‐lapse recordings of Ca2+ transients Video S2. 2D Confocal time‐lapse recordings of Ca2+ transients and t‐tubules [file TJP-597-1833-s001.pptx]

## Slide 1
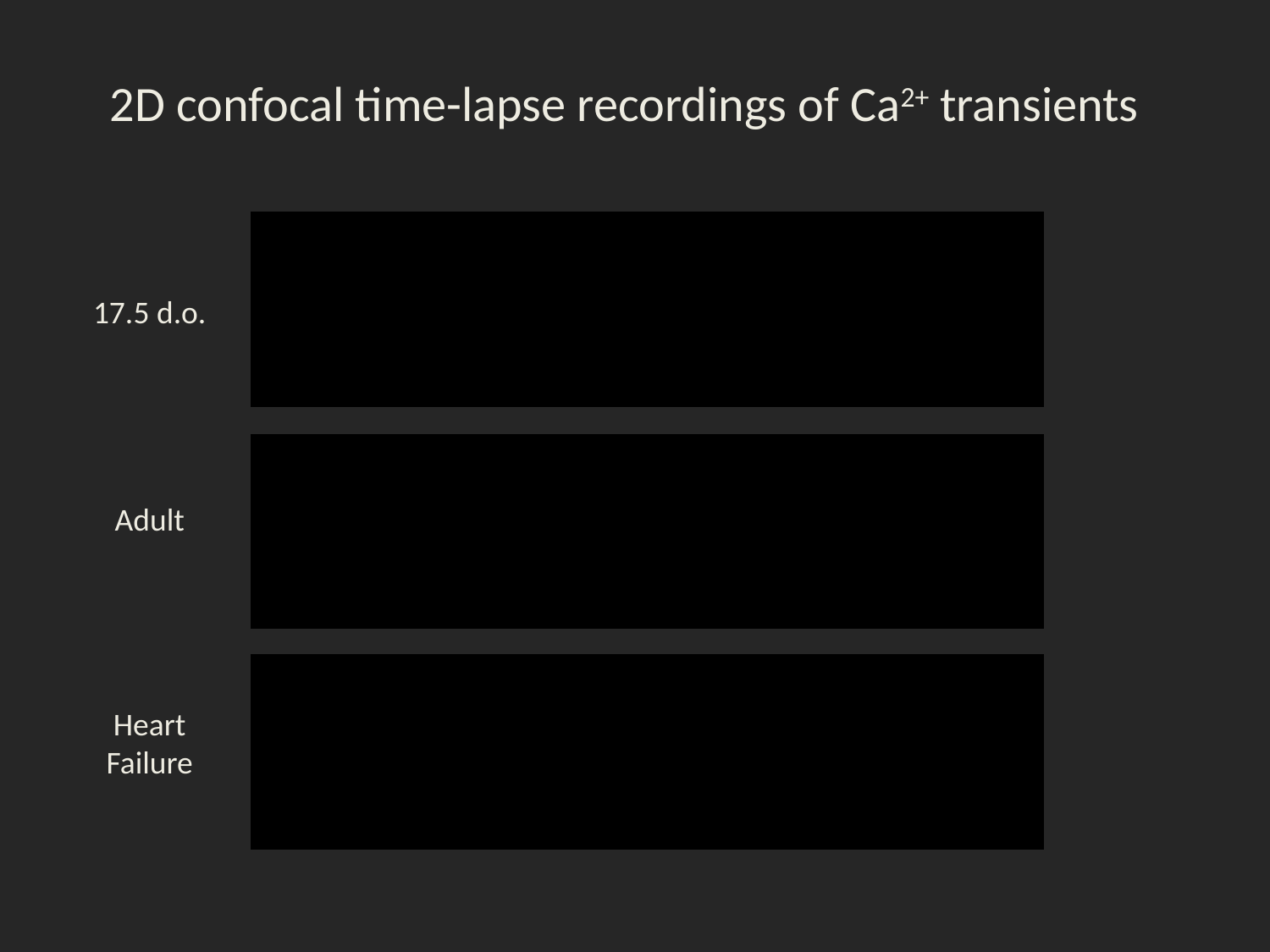

# 2D confocal time-lapse recordings of Ca2+ transients
17.5 d.o.
Adult
Heart
Failure

## Slide 2
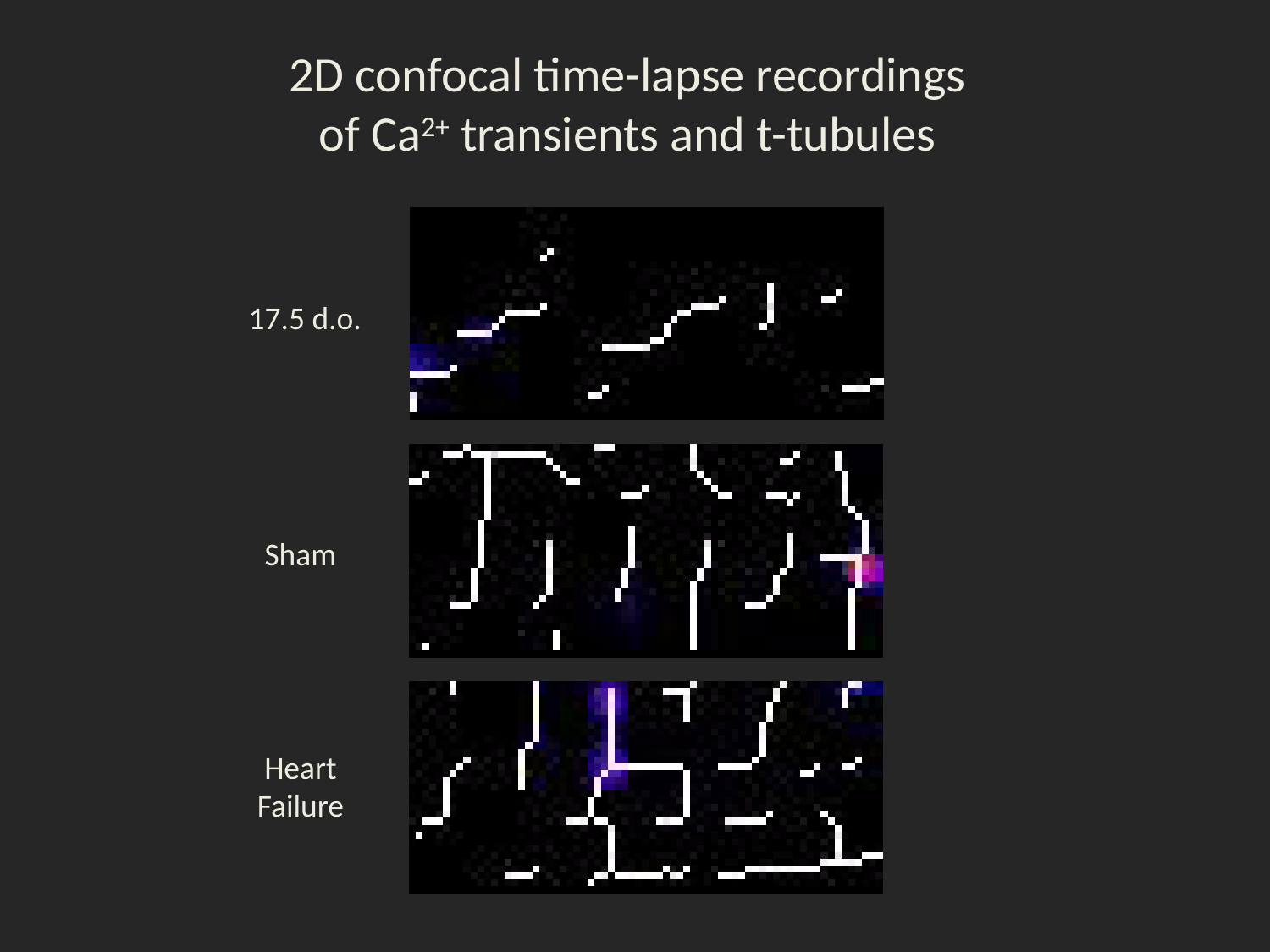

2D confocal time-lapse recordings
of Ca2+ transients and t-tubules
17.5 d.o.
Sham
Heart
Failure
